# Assessing the causal role of immune traits in rheumatoid arthritis by bidirectional Mendelian randomization analysis

**DOI:** 10.18632/aging.205833

**Published:** 2024-05-16

**Authors:** Mingrui Qiu, Huiyun Shen, Weiping Ji, Qiuping Fan

**Affiliations:** 1 Department of Emergency Surgery, Linping Campus, Second Affiliated Hospital, Zhejiang University School of Medicine, Hangzhou 311199, China; 2 Department of Orthopaedic Trauma Surgery, The People’s Hospital of Lishui, Lishui 323000, China

**Keywords:** rheumatoid arthritis, immunity, causal inference, Mendelian randomization

## Abstract

Rheumatoid arthritis (RA) is one of the most common autoimmune joint disorders that leads to cartilage degradation. However, its specific correlation with immune cells has not been thoroughly clarified. Based on the two-sample Mendelian randomization (MR) analysis, the association between RA and 731 immune phenotypes which include morphological parameters (MP), relative cell (RC), median fluorescence intensities (MFI), and absolute cells (AC) was comprehensively determined. After false discovery rate correction, RA and immunophenotypes were statistically associated with each other. It was observed that four immune phenotypes, including 1 MPs, 8 RCs, 15 MFIs, and 10 ACs were causally associated with the risk of RA. Meanwhile, several identified immune traits could serve as independent factors for RA and be robust against pleiotropy. While considering the role of RA in immune traits, the involvement of RA in multiple immunophenotypes including CD62L- myeloid DC AC, CD3 on secreting Treg, CD3 on activated and secreting Treg, and CD3 on CD4 Treg was revealed. This study is the first comprehensive evaluation of the interaction between immune response and RA risk, thus providing therapeutic strategies for RA from an immunological perspective.

## INTRODUCTION

Rheumatoid arthritis (RA) is a prevalent chronic inflammatory and autoimmune disease with global impact. It has been observed to affect the synovial membrane of joints, leading to irreversible cartilage degeneration, bone damage, and premature mortality [1, 2]. The onset and progression of RA are influenced by various pathophysiological factors including environmental and genetic triggers [3]. Recent studies have suggested that immunodeficiency is a characteristic feature of RA and plays a critical role in its early stages [4, 5]. In contrast to osteoarthritis, RA involves the infiltration of several immune cells, such as macrophages, T cells, neutrophils, and B lymphocytes, into the synovial membrane. This infiltration results in the over-proliferation of fibroblast-like synoviocytes and degradation of cartilage [6]. Despite significant advancements in our understanding of RA, there is still a scarcity of meaningful screening targets for its treatment. Therefore, there is an urgent need to elucidate more specific therapeutic targets for RA patients.

Recently, immune cells in synovial membrane have been revealed to be critical in RA occurrence and development. Macrophage and monocyte are two RA severity-connected cells; they can secrete abundant degrading enzymes, chemokines, and cytokines to aggravate joint arthritis formation and bone destruction [7]. The role of T cells has also been abundantly explored in RA. As an anti-inflammation characteristic, CD8+ T cell can delay the progression of RA by reducing the autoimmune responses in rheumatoid joints [8, 9]. Th17 is another type of T cells which contributes to the secretion of granulocyte macrophage colony-stimulating factor in innate lymphocytes and synovial stroma to promote autoimmune arthritis in RA [10]. Nevertheless, the molecular mechanism of these cells in RA have not been thoroughly investigated, and the modulation role of several other immune cells in RA progresses and occurrence still need to be clarified.

In previous studies, CIBERSORT analysis is the most applied method for determining immune cell infiltration level in RA tissues [11, 12], whereas this method is casually influenced by confounding factors and reverse causation in observational studies. Mendelian randomization (MR) is an effective and powerful epidemiological etiology inference analytical method, which makes it possible to determine the correlation between immune trait and diseases more accurately [13, 14]. Recently, MR method has been widely applied in RA pathological analysis, such as causality between RA, osteoporosis [15], cognitive impairment [16], and skin cancers [17], et al. Moreover, numerous studies have proved the validity of MR analysis in assessing the causal relationship of autoimmune diseases, including immune cells [18–20]. Thus, in this study, bi-directional MR analysis was also employed to investigate the causal correlation between731 immune traits and RA, which can greatly improve the understanding of the relationship between two systems and pinpoint helpful insights for RA diagnosis and therapy.

## RESULTS

### Causal effect of immune traits on the risk of RA

**Figure 1:**
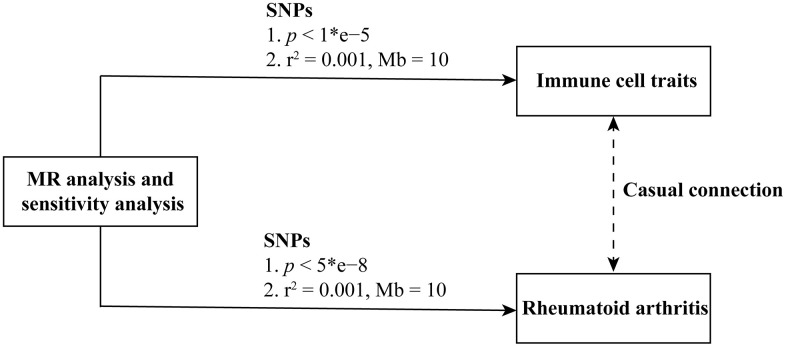
Flowchart of the study.

**Figure 2:**
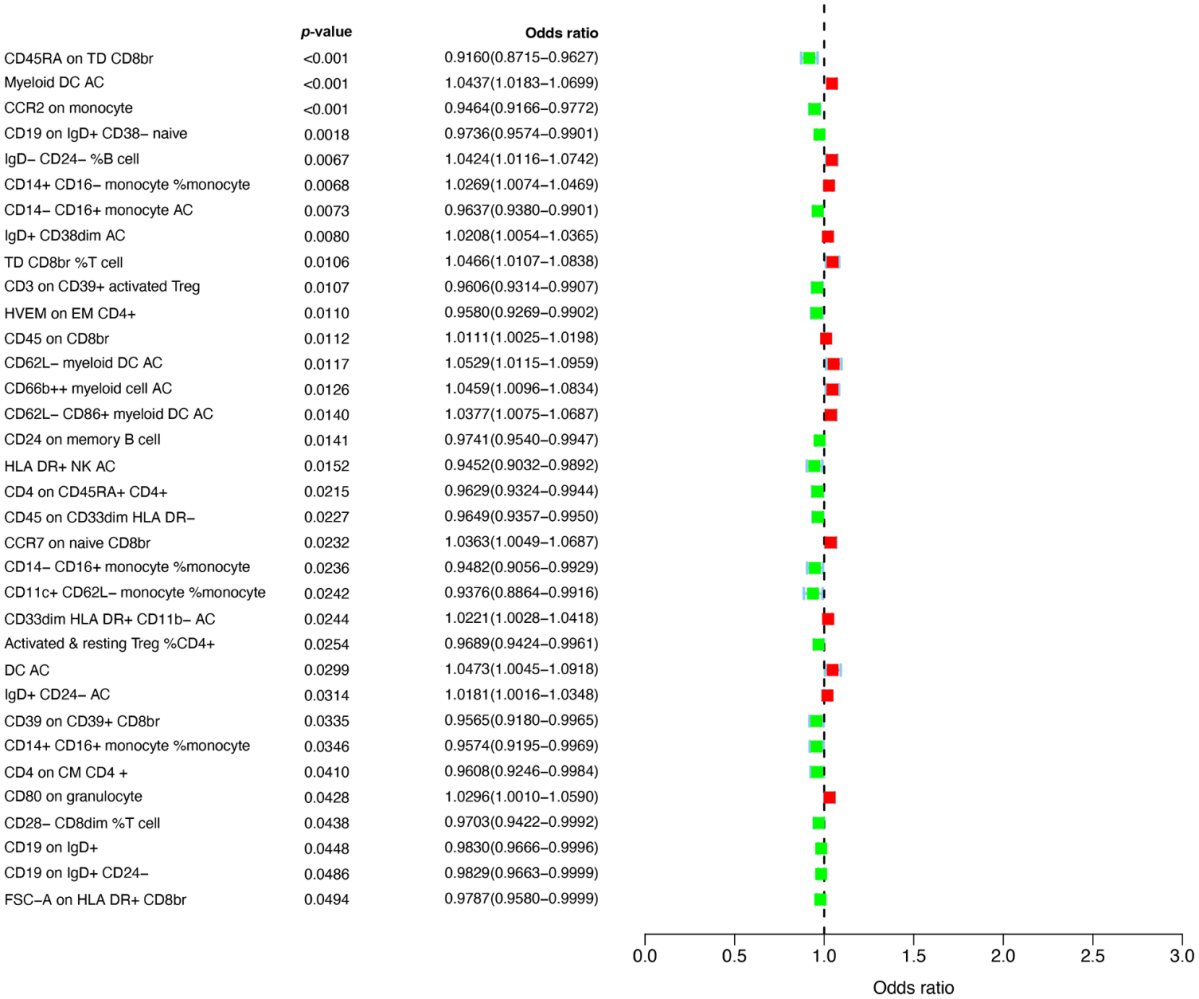
Forest plots showed the causal associations between immune traits and RA.

**Figure 3:**
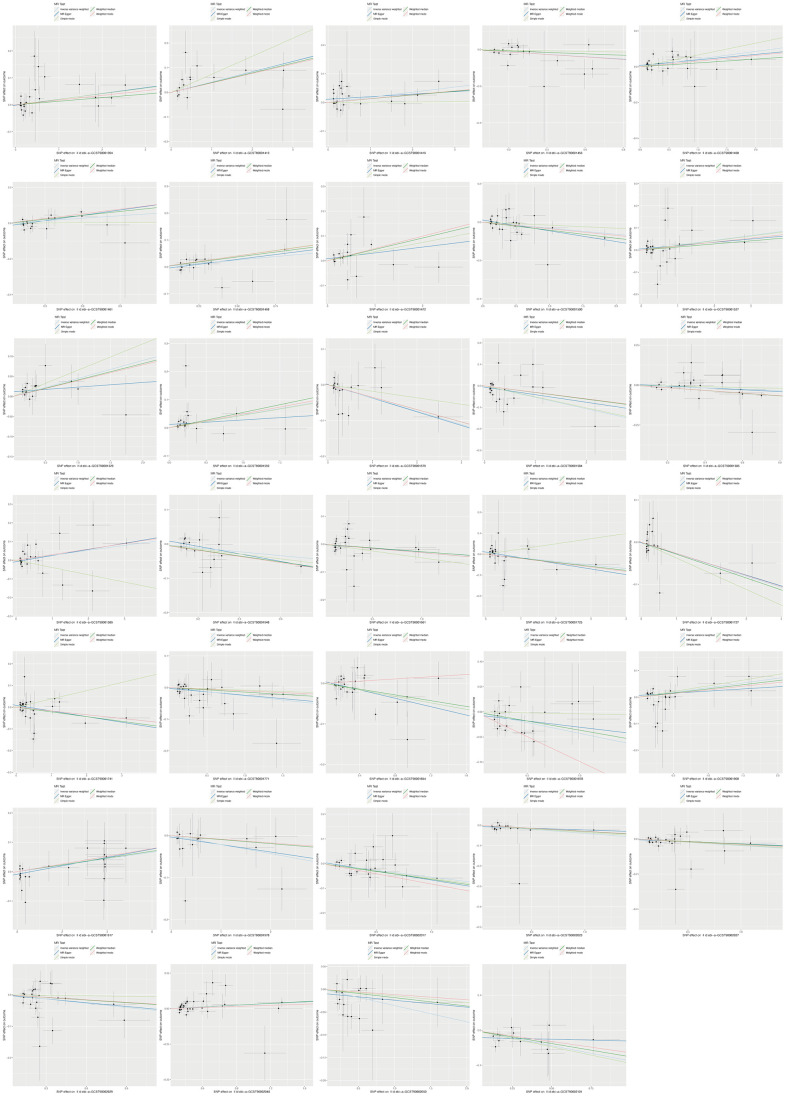
Scatter plots showed the immune traits significantly correlated with RA risk.

The design and flow chat of this study are outlined in Figure 1. Exclude immune traits with significant heterogeneity and pleiotropy, Supplementary Table 1 displayed the IVW results of MR analysis in 34 pairs between immune traits and RA risk which reached a significance level (*p* < 0.05). Given to four classification phenotypes (including MP, RC, MFI, and AC) of immune traits, these RA risk associated immune cells are distributed in 1 MPs, 8 RCs, 15 MFIs, and 10 ACs. Meanwhile, according to the panels of immune trait, 7 RA associated-immune cells are distributed in B cells, 7 belonged to cDC, 6 belonged to Maturation stages of T cell, 4 belonged to monocyte, 3 belonged to myeloid cell, 3 belonged to TBNK, 4 belonged to Treg. The B cell and cDC panel both had the largest number of significant correlation than other panels. Among them, 20 immune traits (including CD45RA on TD CD8br, CD11c^+^ CD62L^-^ monocyte monocyte, HLA DR+ NK AC, CCR2 on monocyte, CD14- CD16+ monocyte monocyte, CD39 on CD39^+^ CD8br, CD14^+^ CD16^+^ monocyte monocyte, HVEM on EM CD4^+^, CD3 on CD39^+^ activated Treg, CD4 on CM CD4^+^, CD4 on CD45RA^+^ CD4^+^, CD14^-^ CD16^+^ monocyte AC, CD45 on CD33dim HLA DR^-^, Activated and resting Treg CD4^+^, CD28^-^ CD8dim T cell, CD19 on IgD^+^ CD38^-^ naive, CD24 on memory B cell, FSC-A on HLA DR^+^ CD8br, CD19 on IgD^+^ CD24^-^, and CD19 on IgD^+^) were negatively correlated with the risk of RA; whereas the other 14 immune traits (including CD45 on CD8br, IgD^+^ CD24^-^ AC, IgD^+^ CD38dim AC, CD33dim HLA DR^+^ CD11b^-^ AC, CD14^+^ CD16^-^ monocyte monocyte, CD80 on granulocyte, CCR7 on naive CD8br, CD62L^-^ CD86^+^ myeloid DC AC, IgD^-^ CD24^-^ B cell, Myeloid DC AC, CD66b^++^ myeloid cell AC, TD CD8br T cell, DC AC, and CD62L^-^ myeloid DC AC) were positively correlated with RA risk according to MR analysis and scatter plots (Figures 2, 3). In addition, CD45RA on TD CD8br trait was discovered as the most causal effect in RA with OR = 0.916, 95% CI = 0.872-0.963, *p* = 5.44 × 10^−4^ (Supplementary Table 2) by IVW analysis; similar results were confirmed in weighted median (OR = 0.927, 95% CI = 0.866-0.992, *p* = 0.029) analysis. The results of pleiotropy analysis indicated there was no significant cross-sectional pleiotropy bias for the effects of selected immune traits on RA (Supplementary Table 3). Moreover, there were also no significant heterogeneities for indicated immune traits effects on RA (Supplementary Table 3), which showed that our MR analysis results of immune traits on RA were quite stability and reliable.

### Causal effect of RA on immunophenotypes

Reverse MR analysis was conducted to explore the causal effects of RA on immune traits. Exclude the result with significant heterogeneity and pleiotropy, RA could significantly influence the risk of 4 immune cells, including CD62L^-^ myeloid DC AC, CD3 on secreting Treg, CD3 on activated and secreting Treg, and CD3 on CD4 Treg (Supplementary Table 4). RA onset could increase the level of CD62L^-^ myeloid DC AC on cDC panel (OR = 1.070, 95% CI = 1.002-1.143, *p* = 0.043); but reduce the level of CD3 on secreting Treg (OR = 0.925, 95% CI = 0.869-0.986, *p* = 0.016), CD3 on activated and secreting Treg (OR = 0.923, 95% CI = 0.866-0.983, *p* = 0.013), and CD3 on CD4 Treg (OR = 0.707, 95% CI = 0.540-0.927, *p* = 0.012) which were all belonged to Treg panel. MR–Egger and Cochrane’s Q value analyses indicated there were no significant heterogeneity and horizontal pleiotropy of RA in indicated immune traits (Supplementary Table 5).

## DISCUSSION

Although it has been widely accepted that the immune system plays a virtual role in RA, there is still a lack of large-scale individual exploration to systematically reveal the correlation between immune cells and the occurrence of RA. GWAS database provide a well opportunity to analysis this relationship from a genetic perspective. Currently, researchers are primarily focused on identifying biomarkers associated with RA. However, none of these biomarkers have been validated through MR analysis. This study is the first to explore the causal correlation between RA and immune traits, aiming to effectively reduce systematic biases (such as reverse causality and confounding factors) that can influence the results of traditional prospective randomized controlled trials. In this study, 34 immune traits in four types of immunophenotypes (1 in MP, 8 in RC, 15 in MFI, and 10 in AC) had significant causal role on RA, among them, RA was also found to act significant causal role on 4 immune traits.

CD45RA on Terminally Differentiated CD8^+^ T cell was presented as the most significant immune trait on RA, and it was proven to be negatively correlated with the risk of RA. According to previous study, the expression of surface molecule CD45 is identified as hub marker of naive T cells differentiated into effector and memory T cells [21]. For example, Lanzavecchia and Sallusto proposed to classify CD4^+^ T cells into 3 subsets based on CD45RA and CCR7 [22]. In such model, the naive T cells are discovered with CD45RA^+^CCR7^+^. Upon antigen encounter, these naïve cells will differentiate into CD45RA^-^CCR7^+^ central memory T cells (TCM), and in further stimulation, these cells can also differentiate into CD45RA^-^CCR7^-^ effector memory T cells (TEM). Compared with TEM is mostly migrated into inflamed samples, TCM subset is accompanied with a high capacity of cell proliferation and IL-2 production [22, 23]. Whereas in CD8^+^ T cells, in addition to having the same 3 subpopulations mentioned above, there are also additional CD45RA^+^CCR7^-^ cell subset. According to previous studies, CD45RA^+^CCR7^-^ CD8^+^ T cells have merely no proliferative capacity and highly sensitive to apoptosis, meanwhile, these cells can express high IFN- and perforin level, but nearly no IL-2 [24, 25]. Thus, CD45RA^+^CCR7^-^ cell subset is presented as the terminally differentiated stage of memory CD8^+^ T cells and named as ultimately effector cells. Moreover, CD45RA^+^CCR7^-^ cells have been regarded as the most effective CD8^+^ T cells for the destruction of cancer cells, and the quality of immune responses to cancers is also usually detected in the light of this cell subset [26]. While considering the role of CD8^+^ T cells in RA, it has been confirmed that CD8^+^ T cells play a virtual role in perpetuation of the inflammatory response and macrophage activation in RA synovial membrane by producing IFN-γ [27]. Meanwhile, it was also found that the peripheral blood from RA patients exhibited a significant increase in CD45RA^–^CD62L^+^ TCM and decrease in CD45RA^+^CD62L^–^ terminally differentiated CD8^+^ T cells, which indicated that the non-antigen-specific effect in RA could promote the differentiation of naïve T cells into central memory T cells; while the skewed phenotype of terminally differentiated CD8^+^ T cells in peripheral RA blood may be owing to the increased migration of these cells to inflammation sites [28, 29]. To explain this phenomenon, we also need to detect this skewed phenotype in RA synovial membrane and fluid. However, long-term follow-up or experimental analysis to determine the content of CD45RA^+^ terminally differentiated CD8^+^ T cells in RA synovial tissues is still extremely lacking.

In addition, it was also noteworthy that the presence of RA was found to be correlated with increased risk of CD62L^-^ myeloid DC AC, and decreased production of CD3 on secreting Treg, CD3 on activated and secreting Treg, and CD3 on CD4 Treg. Treg cells, also named regulatory T cells, are differentiated from the naive CD4^+^T cells. It has been proved that Treg cells play virtual role in immune system suppression by inhibiting the activity of several effector T cells, meanwhile, it can prevent the progression of autoimmune disease by secreting anti-inflammatory chemokines, like IL-10 and TGF-β [30, 31]. As the key immune cells of immune homeostasis, T cells suffer from premature aging in RA patients due to the thymic involution resulted homeostatic proliferation, enormous proliferative stress in antigenic exposure, and their long lifespan [32]. Moreover, the function of Treg cells is also impaired in RA tissues [33]. Repairment of Treg cells might provide effective approach for RA onset and course.

Taken together, two-sample bidirectional MR analysis based on the results of GWAS cohorts was performed to demonstrate the potential correlation between immune traits and RA. Genetic IVs were applied to drive the conclusion of this study, and a variety of MR methods were conducted to analysis the causal inference. Thus, our results are definitely robust, and it provides an important direction for the clinical development of RA. However, there are still several limitations. First, the immune traits and RA datasets are collected at the summary-level, but there is a lack of individual information, so we are unable to conduct population stratification studies for RA patients. Second, the datasets of immune traits and RA come from different ethnic groups, although extensive sensitivity analyses have been performed in this study to reduce the influence of various confounding factors, there is still a certain racial heterogeneity in this MR analysis. Third, in order to assess the correlation between immune traits and RA more comprehensively, a loose threshold SNP screening condition is performed, which may result an increasement of false positive.

In conclusion, this study demonstrates the causal interaction patterns between several immune traits and RA through bidirectional MR analysis. Although its underlying mechanisms for the detected correlation need to be further elicited, the findings still provide novel insights to psychoimmunology of RA onset, and extend the intervention and prevention of RA.

## MATERIALS AND METHODS

### Genome-wide association study (GWAS) data sources

Based on the summary data from GWAS database, two-sample MR analysis was used to evaluate the bidirectional causal correlation between 731 immune traits (including 7 immune panels) and RA. The GWAS data for 731 immune traits were acquired from the GWAS Catalog from GCST90001391 to GCST90002121 which include 32 morphological parameters (MP), 192 relative cell (RC) counts, 389 median fluorescence intensities (MFI) reflecting surface antigen levels, and 118 absolute cells (AC) counts [13]. A total of 3,757 Sardinian normal cases were performed in this study. After adjusting for covariates age^2^, age, and sex, approximately 22 million single nucleotide polymorphisms (SNPs) genotyped with high-density arrays were conducted with Sardinian sequence-based reference panel [34]. The GWAS data for RA (*N*_case_ = 12,555, *N*_control_ = 240,862) were publicly available from the FinnGen database which includes 500,000 Finnish biobank individuals, and independent genomic loci were identified by comparing with healthy control.

### Instrumental variables (IVs) selection for MR analysis

Filtering steps were performed for each immune trait and RA dataset to meet valid IVs according to previous research [13, 35]. For immune traits, the significance thresholds were set to 1 × 10^−5^, and then PLINK software was applied to extract the significant and independent SNPs with linkage disequilibrium r^2^ = 0.001 within 10 Mb distance [19, 20, 36]. A total of 7 to 1,786 independent immunophenotype IVs were selected for further analysis. For RA, the genome-wide significance thresholds were adjusted to 5 × 10^−8^, and the linkage disequilibrium was adjusted to r^2^ = 0.001 within 10 Mb distance [37]. In addition, to avoid the weak instrumental bias of MR analysis, the proportion of phenotypic variation explained and F-statistic were also calculated to test the robustness of SNPs, and SNPs with F-statistics < 10 were removed from this study. Ultimately, 12 SNPs were screened out when using RA as exposure factors for further reverse direction MR analysis.

### Statistical analysis

The “TwoSampleMR” R package was performed to estimate the correlation between immune traits and the risk of RA. Robust analytical methods, including weighted median, inverse variance weighting (IVW), mode-based methods, MR pleiotropy residual sum and outlier (MR-PRESSO), and MR Egger were mainly employed. Among them, the IVW analysis was used as the primary method in this study, and Cochran’s Q statistic test and funneled plot was utilized to examine the heterogeneity of SNPs. If there was possible heterogeneity within SNPs which was indicated by the rejected null hypothesis, the random-effect IVW was applied instead of the fixed-effect IVW [38]. Pleiotropy is another outlier that could substantially affect the results of estimation. Thus, we also applied MR Egger and MR-PRESSO regression analyses to exclude possible bias resulted by horizontal pleiotropy [39, 40]. The main MR analysis methods were also repeated to examine the causal role of RA on immune traits.

### Availability of data and materials

The datasets used and/or analyzed during the current study are available from the corresponding author on reasonable request.

## Supplementary Material

Supplementary Table 1

Supplementary Table 2

Supplementary Table 3

Supplementary Table 4

Supplementary Table 5

## References

[1] Smolen JS, Aletaha D, McInnes IB. Rheumatoid arthritis. Lancet. 2016; 388:2023–38. 10.1016/S0140-6736(16)30173-8 27156434

[2] Ge X, Frank-Bertoncelj M, Klein K, McGovern A, Kuret T, Houtman M, Burja B, Micheroli R, Shi C, Marks M, Filer A, Buckley CD, Orozco G, et al. Functional genomics atlas of synovial fibroblasts defining rheumatoid arthritis heritability. Genome Biol. 2021; 22:247. 10.1186/s13059-021-02460-6 34433485 PMC8385949

[3] Klareskog L, Padyukov L, Lorentzen J, Alfredsson L. Mechanisms of disease: Genetic susceptibility and environmental triggers in the development of rheumatoid arthritis. Nat Clin Pract Rheumatol. 2006; 2:425–33. 10.1038/ncprheum0249 16932734

[4] Katsuragi T, Iwashige A, Tsukada J. [Immunodeficiency-associated Burkitt lymphoma developed in a patient receiving a long-term methotrexate therapy for rheumatoid arthritis]. Rinsho Ketsueki. 2016; 57:9–14. 10.11406/rinketsu.57.9 26861097

[5] Guo Z, Ma Y, Wang Y, Xiang H, Cui H, Fan Z, Zhu Y, Xing D, Chen B, Tao H, Guo Z, Wu X. Identification and validation of metabolism-related genes signature and immune infiltration landscape of rheumatoid arthritis based on machine learning. Aging (Albany NY). 2023; 15:3807–25. 10.18632/aging.204714 37166429 PMC10449312

[6] Bustamante MF, Garcia-Carbonell R, Whisenant KD, Guma M. Fibroblast-like synoviocyte metabolism in the pathogenesis of rheumatoid arthritis. Arthritis Res Ther. 2017; 19:110. 10.1186/s13075-017-1303-3 28569176 PMC5452638

[7] Elshabrawy HA, Chen Z, Volin MV, Ravella S, Virupannavar S, Shahrara S. The pathogenic role of angiogenesis in rheumatoid arthritis. Angiogenesis. 2015; 18:433–48. 10.1007/s10456-015-9477-2 26198292 PMC4879881

[8] Carvalheiro H, da Silva JA, Souto-Carneiro MM. Potential roles for CD8(+) T cells in rheumatoid arthritis. Autoimmun Rev. 2013; 12:401–9. 10.1016/j.autrev.2012.07.011 22841983

[9] Higashioka K, Yoshimura M, Sakuragi T, Ayano M, Kimoto Y, Mitoma H, Ono N, Arinobu Y, Kikukawa M, Yamada H, Horiuchi T, Akashi K, Niiro H. Human PD-1^hi^CD8^+^ T Cells Are a Cellular Source of IL-21 in Rheumatoid Arthritis. Front Immunol. 2021; 12:654623. 10.3389/fimmu.2021.654623 33815416 PMC8017303

[10] Hirota K, Hashimoto M, Ito Y, Matsuura M, Ito H, Tanaka M, Watanabe H, Kondoh G, Tanaka A, Yasuda K, Kopf M, Potocnik AJ, Stockinger B, et al. Autoimmune Th17 Cells Induced Synovial Stromal and Innate Lymphoid Cell Secretion of the Cytokine GM-CSF to Initiate and Augment Autoimmune Arthritis. Immunity. 2018; 48:1220–32.e5. 10.1016/j.immuni.2018.04.009 29802020 PMC6024031

[11] Zhou S, Lu H, Xiong M. Identifying Immune Cell Infiltration and Effective Diagnostic Biomarkers in Rheumatoid Arthritis by Bioinformatics Analysis. Front Immunol. 2021; 12:726747. 10.3389/fimmu.2021.726747 34484236 PMC8411707

[12] Wang J, Xue Y, Zhou L. Comparison of immune cells and diagnostic markers between spondyloarthritis and rheumatoid arthritis by bioinformatics analysis. J Transl Med. 2022; 20:196. 10.1186/s12967-022-03390-y 35509008 PMC9066892

[13] Orrù V, Steri M, Sidore C, Marongiu M, Serra V, Olla S, Sole G, Lai S, Dei M, Mulas A, Virdis F, Piras MG, Lobina M, et al. Complex genetic signatures in immune cells underlie autoimmunity and inform therapy. Nat Genet. 2020; 52:1036–45. 10.1038/s41588-020-0684-4 32929287 PMC8517961

[14] Bentham J, Morris DL, Graham DS, Pinder CL, Tombleson P, Behrens TW, Martín J, Fairfax BP, Knight JC, Chen L, Replogle J, Syvänen AC, Rönnblom L, et al. Genetic association analyses implicate aberrant regulation of innate and adaptive immunity genes in the pathogenesis of systemic lupus erythematosus. Nat Genet. 2015; 47:1457–64. 10.1038/ng.3434 26502338 PMC4668589

[15] Liu YQ, Liu Y, Chen ZY, Li H, Xiao T. Rheumatoid arthritis and osteoporosis: a bi-directional Mendelian randomization study. Aging (Albany NY). 2021; 13:14109–30. 10.18632/aging.203029 34015765 PMC8202858

[16] Duan L, Li S, Li H, Shi Y, Xie X, Feng Y. Causality between rheumatoid arthritis and the risk of cognitive impairment: a Mendelian randomization study. Arthritis Res Ther. 2024; 26:5. 10.1186/s13075-023-03245-x 38167504 PMC10759661

[17] Yu N, Qi H, Guo Y, Wu L, Su J, Huang K, Li Y, Jiang Z, Zhao S, Chen X. Associations between rheumatoid arthritis and skin cancer: A bidirectional two-sample Mendelian randomization study. J Am Acad Dermatol. 2024; 90:198–200. 10.1016/j.jaad.2023.09.046 37758025

[18] Yu XH, Cao RR, Yang YQ, Lei SF. Identification of causal metabolites related to multiple autoimmune diseases. Hum Mol Genet. 2022; 31:604–13. 10.1093/hmg/ddab273 34523675

[19] Wang C, Zhu D, Zhang D, Zuo X, Yao L, Liu T, Ge X, He C, Zhou Y, Shen Z. Causal role of immune cells in schizophrenia: Mendelian randomization (MR) study. BMC Psychiatry. 2023; 23:590. 10.1186/s12888-023-05081-4 37582716 PMC10428653

[20] Cao RR, Yu XH, Xiong MF, Li XT, Deng FY, Lei SF. The immune factors have complex causal regulation effects on bone mineral density. Front Immunol. 2022; 13:959417. 10.3389/fimmu.2022.959417 36341399 PMC9630477

[21] Sallusto F, Kremmer E, Palermo B, Hoy A, Ponath P, Qin S, Förster R, Lipp M, Lanzavecchia A. Switch in chemokine receptor expression upon TCR stimulation reveals novel homing potential for recently activated T cells. Eur J Immunol. 1999; 29:2037–45. 10.1002/(SICI)1521-4141(199906)29:06<2037::AID-IMMU2037>3.0.CO;2-V 10382767

[22] Lanzavecchia A, Sallusto F. Dynamics of T lymphocyte responses: intermediates, effectors, and memory cells. Science. 2000; 290:92–7. 10.1126/science.290.5489.92 11021806

[23] Sallusto F, Lenig D, Förster R, Lipp M, Lanzavecchia A. Two subsets of memory T lymphocytes with distinct homing potentials and effector functions. Nature. 1999; 401:708–12. 10.1038/44385 10537110

[24] Geginat J, Lanzavecchia A, Sallusto F. Proliferation and differentiation potential of human CD8+ memory T-cell subsets in response to antigen or homeostatic cytokines. Blood. 2003; 101:4260–6. 10.1182/blood-2002-11-3577 12576317

[25] Champagne P, Ogg GS, King AS, Knabenhans C, Ellefsen K, Nobile M, Appay V, Rizzardi GP, Fleury S, Lipp M, Förster R, Rowland-Jones S, Sékaly RP, et al. Skewed maturation of memory HIV-specific CD8 T lymphocytes. Nature. 2001; 410:106–11. 10.1038/35065118 11242051

[26] Carrasco J, Godelaine D, Van Pel A, Boon T, van der Bruggen P. CD45RA on human CD8 T cells is sensitive to the time elapsed since the last antigenic stimulation. Blood. 2006; 108:2897–905. 10.1182/blood-2005-11-007237 16857986

[27] Mortarini R, Piris A, Maurichi A, Molla A, Bersani I, Bono A, Bartoli C, Santinami M, Lombardo C, Ravagnani F, Cascinelli N, Parmiani G, Anichini A. Lack of terminally differentiated tumor-specific CD8+ T cells at tumor site in spite of antitumor immunity to self-antigens in human metastatic melanoma. Cancer Res. 2003; 63:2535–45. 12750277

[28] Maldonado A, Mueller YM, Thomas P, Bojczuk P, O’Connors C, Katsikis PD. Decreased effector memory CD45RA+ CD62L- CD8+ T cells and increased central memory CD45RA- CD62L+ CD8+ T cells in peripheral blood of rheumatoid arthritis patients. Arthritis Res Ther. 2003; 5:R91–6. 10.1186/ar619 12718752 PMC165030

[29] Scarsi M, Ziglioli T, Airò P. Decreased circulating CD28-negative T cells in patients with rheumatoid arthritis treated with abatacept are correlated with clinical response. J Rheumatol. 2010; 37:911–6. 10.3899/jrheum.091176 20231200

[30] Corthay A. How do regulatory T cells work? Scand J Immunol. 2009; 70:326–36. 10.1111/j.1365-3083.2009.02308.x 19751267 PMC2784904

[31] Gautam S, Kumar R, Kumar U, Kumar S, Luthra K, Dada R. Yoga maintains Th17/Treg cell homeostasis and reduces the rate of T cell aging in rheumatoid arthritis: a randomized controlled trial. Sci Rep. 2023; 13:14924. 10.1038/s41598-023-42231-w 37696876 PMC10495372

[32] Goronzy JJ, Weyand CM. Mechanisms underlying T cell ageing. Nat Rev Immunol. 2019; 19:573–83. 10.1038/s41577-019-0180-1 31186548 PMC7584388

[33] Jiang Q, Yang G, Liu Q, Wang S, Cui D. Function and Role of Regulatory T Cells in Rheumatoid Arthritis. Front Immunol. 2021; 12:626193. 10.3389/fimmu.2021.626193 33868244 PMC8047316

[34] Sidore C, Busonero F, Maschio A, Porcu E, Naitza S, Zoledziewska M, Mulas A, Pistis G, Steri M, Danjou F, Kwong A, Ortega Del Vecchyo VD, Chiang CW, et al. Genome sequencing elucidates Sardinian genetic architecture and augments association analyses for lipid and blood inflammatory markers. Nat Genet. 2015; 47:1272–81. 10.1038/ng.3368 26366554 PMC4627508

[35] Yu XH, Yang YQ, Cao RR, Bo L, Lei SF. The causal role of gut microbiota in development of osteoarthritis. Osteoarthritis Cartilage. 2021; 29:1741–50. 10.1016/j.joca.2021.08.003 34425228

[36] Gu J, Yan GM, Kong XL, Zhang YY, Huang LH, Lu HM. Assessing the causal relationship between immune traits and systemic lupus erythematosus by bi-directional Mendelian randomization analysis. Mol Genet Genomics. 2023; 298:1493–503. 10.1007/s00438-023-02071-9 37845373

[37] Wei D, Jiang Y, Cheng J, Wang H, Sha K, Zhao J. Assessing the association of leukocyte telomere length with ankylosing spondylitis and rheumatoid arthritis: A bidirectional Mendelian randomization study. Front Immunol. 2023; 14:1023991. 10.3389/fimmu.2023.1023991 37033949 PMC10080099

[38] Burgess S, Small DS, Thompson SG. A review of instrumental variable estimators for Mendelian randomization. Stat Methods Med Res. 2017; 26:2333–55. 10.1177/0962280215597579 26282889 PMC5642006

[39] Burgess S, Thompson SG. Interpreting findings from Mendelian randomization using the MR-Egger method. Eur J Epidemiol. 2017; 32:377–89. 10.1007/s10654-017-0255-x 28527048 PMC5506233

[40] Verbanck M, Chen CY, Neale B, Do R. Detection of widespread horizontal pleiotropy in causal relationships inferred from Mendelian randomization between complex traits and diseases. Nat Genet. 2018; 50:693–8. 10.1038/s41588-018-0099-7 29686387 PMC6083837

